# The effects of ischaemic conditioning on lung ischaemia–reperfusion injury

**DOI:** 10.1186/s12931-022-02288-z

**Published:** 2022-12-16

**Authors:** Dimitrios Vlastos, Mohamed Zeinah, George Ninkovic-Hall, Stefanos Vlachos, Agni Salem, Athanasios Asonitis, Hemangi Chavan, Lazaros Kalampalikis, Abdullah Al Shammari, José María Alvarez Gallesio, Aina Pons, Ioanna Andreadou, Ignatios Ikonomidis

**Affiliations:** 1 Department of Vascular Surgery, Countess of Chester Hospital, Chester, UK; 2 Second Department of Cardiology, Attikon University Hospital, Athens, Greece; 3 Department of Cardiac Surgery, Liverpool Heart and Chest Hospital, Liverpool, UK; 4 Ain Shams University, Cairo, Egypt; 5 Department of Vascular Surgery, Royal Liverpool University Hospital, Liverpool, UK; 6 Department of Cardiothoracic Surgery, NHS Golden Jubilee National Hospital, Glascow, UK; 7 Department of Thoracic Surgery, Royal Brompton and Harefield NHS Foundation Trust, London, UK; 8 Department of Minimally Invasive Cardiac Surgery, Metropolitan General Hospital, Athens, Greece; 9 School of Pharmacy, National and Kapodistrian University of Athens, Athens, Greece; 10 Present Address: Liverpool, UK

**Keywords:** Lung ischaemia–reperfusion, Ischaemic conditioning, Ischaemia–reperfusion injury, Acute lung injury, Acute respiratory distress syndrome

## Abstract

Ischaemia–reperfusion injury (IRI) encompasses the deleterious effects on cellular function and survival that result from the restoration of organ perfusion. Despite their unique tolerance to ischaemia and hypoxia, afforded by their dual (pulmonary and bronchial) circulation as well as direct oxygen diffusion from the airways, lungs are particularly susceptible to IRI (LIRI). LIRI may be observed in a variety of clinical settings, including lung transplantation, lung resections, cardiopulmonary bypass during cardiac surgery, aortic cross-clamping for abdominal aortic aneurysm repair, as well as tourniquet application for orthopaedic operations. It is a diagnosis of exclusion, manifesting clinically as acute lung injury (ALI) or acute respiratory distress syndrome (ARDS). Ischaemic conditioning (IC) signifies the original paradigm of treating IRI. It entails the application of short, non-lethal ischemia and reperfusion manoeuvres to an organ, tissue, or arterial territory, which activates mechanisms that reduce IRI. Interestingly, there is accumulating experimental and preliminary clinical evidence that IC may ameliorate LIRI in various pathophysiological contexts. Considering the detrimental effects of LIRI, ranging from ALI following lung resections to primary graft dysfunction (PGD) after lung transplantation, the association of these entities with adverse outcomes, as well as the paucity of protective or therapeutic interventions, IC holds promise as a safe and effective strategy to protect the lung. This article aims to provide a narrative review of the existing experimental and clinical evidence regarding the effects of IC on LIRI and prompt further investigation to refine its clinical application.

## Background

Ischaemia–reperfusion injury (IRI) encompasses the deleterious effects on cellular function and survival that result from the restoration of organ perfusion [1]. Counterintuitively, IRI further aggravates ischaemic organ damage as the degree of injury after reperfusion surpasses that caused by ischaemia per se [2]. It is mediated by sterile inflammation, enhanced oxidative stress and coagulation, endothelial dysfunction, and activation of cellular death pathways [1, 2]. Crucially, it is a systemic process with the potential to evoke distant organ injury and progress to multiple organ dysfunction syndrome [2]. Despite their unique tolerance to ischaemia and hypoxia, afforded by their dual (pulmonary and bronchial) circulation as well as direct oxygen diffusion from the airways [3], lungs are particularly susceptible to IRI (LIRI) [4]. Importantly, the cessation of ventilation leads to functional impairments similar to those induced by hypoperfusion [3], to which it is also interrelated by way of hypoxic pulmonary vasoconstriction (HPV) [5]. LIRI may be observed in a variety of clinical settings, including lung transplantation [4, 6], lung resections [7, 8], cardiopulmonary bypass (CPB) during cardiac surgery [9–12], aortic cross-clamping for abdominal aortic aneurysm (AAA) repair [13], as well as tourniquet application for orthopaedic operations [14]. It culminates in the breakdown of lung endothelial and epithelial barriers, leading to pulmonary oedema with attendant gas exchange impairment [4] and increased pulmonary vascular resistance resulting in pulmonary hypertension [15].

Ischaemic conditioning (IC) signifies the original paradigm of treating IRI. It entails the application of short, non-lethal ischemia and reperfusion manoeuvres to an organ, tissue, or arterial territory, which activates mechanisms that reduce IRI [16]. The concept of IC has several temporal and anatomical variations. In specific, the protective ischaemic stimulus may be applied before, during, or following the index reperfusion episode (pre-, per-, and post-conditioning respectively) with similar beneficial effects [16]. Furthermore, this intervention has systemic protective properties which are exploited by remote ischemic conditioning (RIC): biochemical and neuronal mechanisms confer protection to organs distant to the conditioning stimulus [16]. There is accumulating experimental and clinical evidence that IC may ameliorate LIRI in various pathophysiological contexts. In specific, it reduces the underlying oxidative stress and sterile inflammation in animal [17] and human models [7]. This is translated into decreased histologic damage [18], reduced pulmonary oedema [19], and improved respiratory function [8, 19] and pulmonary vascular haemodynamics [10, 20]. Considering the detrimental effects of LIRI, ranging from acute lung injury (ALI) following lung resections [15] to primary graft dysfunction (PGD) after lung transplantation [4], the association of these entities with adverse outcomes [4, 21], as well as the paucity of protective or therapeutic clinical interventions [4], ischaemic conditioning holds promise as a safe and effective strategy to protect the lung. This article aims to provide a review of the existing experimental and clinical evidence regarding the effects of IC on LIRI.

## The pathophysiology of lung ischaemia reperfusion injury

**Fig. 1:**
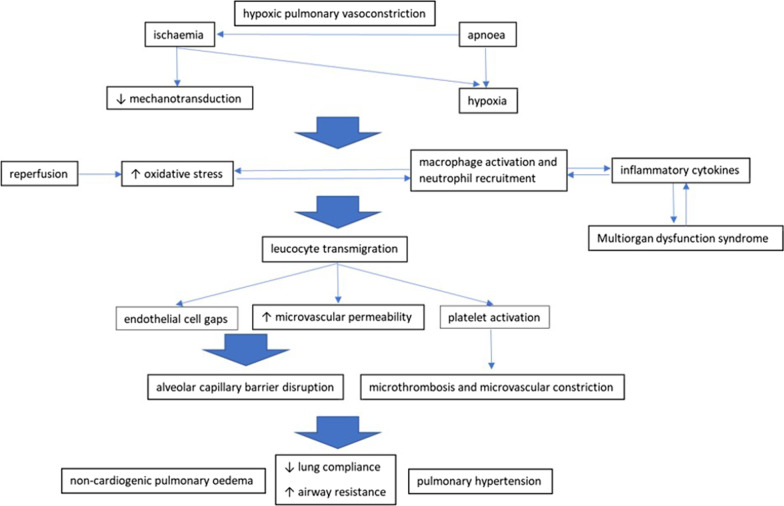
The pathophysiology of lung ischaemia reperfusion injury. Lung ischaemia reperfusion injury is the result of a robust inflammatory response and oxidative stress enhancement, which are instigated by ischaemia and further promoted by reperfusion. The attendant leucocyte migration and platelet activation result in alveolar-epithelial barrier impairment and pulmonary vasoconstriction, manifesting as noncardiogenic pulmonary oedema, pulmonary hypertension and deterioration of ventilation mechanics

Enhanced oxidative stress appears to play a prominent role in the pathophysiology of LIRI [22]. Ischaemia, in the presence or not of apnoea as determined by ventilatory manoeuvres, creates a hypoxic environment with inhibited mechanotransduction in the arterioles and capillaries [23]. This triggers the production of reactive oxygen species (ROS) by endothelial cells, macrophages, and other immune cells [24]. The lung antioxidative mechanisms are overwhelmed upon reperfusion, resulting in an imbalance between ROS production and clearance [22]. Eventually, direct oxidative damage ensues with carbonylation of proteins and peroxynitration of proteins, lipids, and DNA [25]. Furthermore, ROS instigate a robust innate immune response by activating alveolar macrophages, which in turn release proinflammatory cytokines, including interleukin (IL)-8, -12, 18, and tumour necrosis factor (TNF)-a [26]. Additionally, neutrophils are recruited [26], and in concert with macrophages further enhance ROS generation; thus, a self-perpetuating cycle of oxidative stress enhancement is created [27, 28]. These activated leucocytes transmigrate into the extravascular space and cause increased microvascular permeability, thrombosis, oedema, and parenchymal cell death, by way of proteases, elastases, and ROS production [2]. Adherence of neutrophils to the endothelium further promotes the formation of gaps between the endothelial cells [6]. Moreover, these inflammatory cascades trigger platelet aggregation and coagulation, leading to formation of microthrombi and microvascular constriction [29]. The attendant activation of vasoactive agents, including thromboxane A2 and platelet activating factor, further promotes oedema formation [29, 30]. LIRI is also characterised by apoptotic phenomena, in part initiated by inflammatory cytokines (including IL-1β, -2, -8, and TNF-a) and enhanced by the release of proapoptotic factors due to mitochondrial rupture [31]. More specifically, the hypoxia-induced adenosine triphosphate (ATP) deficiency induces dysfunction of ATP-dependent ion pumps that results in mitochondrial calcium overload [29]. This leads to increased permeability of the mitochondrial transition pores and swelling, eventually leading to rupture [31]. Type II epithelial cells play a key role as both victim and as culprit of LIRI, as their reperfusion-induced dysfunction leads to impaired production and composition of pulmonary surfactant [32]. Crucially, the above described prooxidative and inflammatory signalling may exert systemic effects: on the one hand, distant IRI is known to cause pulmonary injury [33]; on the other hand, LIRI induces remote organ inflammatory and oxidative damage [34], while unilateral lung reperfusion may lead to similar deleterious processes in the contralateral, non-ischaemic lung [35] (Fig. 1).

## The clinical impact of lung ischaemia reperfusion injury

The end result of LIRI is disruption of the alveolar-capillary barrier, causing non-cardiogenic pulmonary oedema and ventilation-perfusion (V/Q) [5]. Total and extravascular lung water is increased, causing gas exchange and lung mechanics impairment, with decreased arterial oxygen tension (PaO2) [36], increased airway pressures and increased alveolar-arterial oxygen gradient [(A-a) DO2] [37]. In addition, the attendant defective surfactant production and composition leads to reduced static (Cs) and dynamic lung compliance (Cd), while further increasing (A-a) DO2 [38]. Moreover, pulmonary vascular resistance (PVR) is increased up to three-fold following reperfusion, mainly due to pulmonary precapillary vasoconstriction [39]. Thus, LIRI is further compounded by pulmonary hypertension, which might also additionally hydrostatically promote the formation of pulmonary oedema [40].

In the absence of specific diagnostic criteria, LIRI is a diagnosis of exclusion, manifesting clinically as ALI or acute respiratory distress syndrome (ARDS) [41]. It has a detrimental impact in various clinical scenarios, particularly following lung transplantation: LIRI leads to PGD, which is the major cause of both short- and long-term morbidity and mortality in this setting [15, 42]. Importantly, the incidence of severe (grade 3) PGD within 72 h of transplantation is approximately 30% [42], while PGD is also associated with late graft rejection, which is the primary mortality aetiology beyond 1 year of the procedure [43, 44]. ALI/ARDS is frequent following lung resections, with a reported incidence of 0.88%, 2.96%, and 7.9% following sublobar, lobar/bilobar resection, and pneumonectomy respectively [21]. The associated mortality is considerable, ranging from 22% in sublobar resections to 50% following pneumonectomy [21]. LIRI is also a major source of morbidity and mortality after cardiac surgery, as it contributes to the development of ARDS, with an incidence of up to 20% and a mortality reaching 80% [45]. Interestingly, hepatosplanchnic IRI in aortic surgery has been found to cause lung injury, with increased pulmonary leak index (PLI) in up to 74% of the patients, especially in cases involving clamping of major aortic branches and in direct correlation with the aortic clamping time [46]. ALI also complicates 30–50% of major trauma cases, with an associated mortality of 10% depending on the severity of lung dysfunction [47].

## The protective effects of ischemic conditioning on lung ischaemia reperfusion injury—effects on oxidative stress and inflammation

### Experimental evidence

One of the critical mechanisms mediating the protective effects of IC against LIRI is the alleviation of oxidative stress and inflammation. Li et al*.* were among the first to demonstrate this, in a canine model of lung transplantation (in situ LIRI). Specifically, IC by donor pulmonary hilar occlusion/reperfusion before transplantation was associated with reduced infiltration of the transplanted lung interstitium by polymorphonuclear leukocytes (PMNs) and reduced blood levels of malondialdehyde (MDA) and thromboxane B2 (TXB2) following reperfusion; on the contrary, superoxide dismutase (SOD) levels were higher, denoting a preserved antioxidant reserve [48]. Similarly, in a rat model experiment, IC decreased thiobarbituric acid reactive substances (TBARS) after lung storage, reflecting the decreased MDA levels [49]. Focusing on the fluid shifts caused by enhanced oxidative stress and inflammation, Gasparri et al. showed that 15, but not 5, minutes of transient lung ischaemia before lung preservation mitigated lung oedema upon reperfusion [50]. Soncul et al*.* utilised isolated lungs mounted on a modified Langendorff perfusion apparatus, where IC was related with reduced tissue and perfusate MDA levels [51]. In a similar IC protocol, tissue MDA levels were reduced, glutathione levels increased, intra-alveolar oedema and capillary congestion were prevented, while type II alveolar and endothelial cells were preserved [52]. Li et al*.* applied IC before sustained lung ischemia–reperfusion to rabbits. Lung MDA levels were lower and SOD levels were higher in the preconditioned lungs, with an attendant reduction in lung oedema and alveolar damage [53]. Friedrich et al*.* applied IC on a canine model of LIRI, whereby the reperfusion insult was preceded by either a single 5-min occlusion with a 15-min reperfusion period or two successive 10-min ischemia–reperfusion stimuli, and was followed by bronchoalveolar lavage (BAL). Interestingly, the former protocol resulted in reduced BAL fluid protein and TNF-a content, while the latter had no effect, highlighting the importance of the IC stimulus duration [54]. Jun et al*.* elucidated the genetic background of IC: conditioning of the donor lung resulted in downregulation of a vast array of inflammatory and immune mediator genes, including IL-1, IL-2, IL-3, IL-6, IL-15, TNF-a, intercellular adhesion molecule-2 (ICAM-2), vascular cell adhesion molecule-1 (VCAM-1), and activated leukocyte adhesion molecules [55].

Remote ischemic conditioning (RIC) appears to have similar effects to local IC on in situ LIRI, as demonstrated by Song et al.: 6 cycles of 10-s aortic occlusion/reperfusion protected from LIRI induced by lung hilar clamping. In more detail, alveoli were preserved with less neutrophilic infiltration and oedema, the increase in lung wet-to-dry weight ratio was prevented, while plasma IL-6, TNF-a, and ROS levels were reduced [17]. Waldow et al*.* similarly concluded that RIC prevented the IL-1β increase and abrogated the lung macrophage infiltration induced by LIRI [20]. RIC by hepatic hilar clamping also ameliorated the increase in IL-6 and TNF-a, reduced myeloperoxidase (MPO) activity (reflecting the respective lung neutrophil accumulation) and the BAL fluid leucocyte levels, in parallel with inhibited alveolar damage and reduced wet-to-dry lung ratio; apoptotic cascades were attenuated, with decreased cleaved caspase-3 expression and fewer apoptotic nuclei [19]. An interesting study by Zhou et al*.* highlighted the protection conferred against CPB-induced LIRI: RIC alleviated intra-alveolar neutrophil infiltration and alveolar wall thickening, reduced BAL fluid protein levels and lung wet-to-dry weight ratio, in parallel with increased anti-inflammatory IL-4 and IL-10 [56].

Besides in situ LIRI, conditioning exerts protection in cases of remote organ reperfusion. In the setting of aortic occlusion and reperfusion, RIC was associated with reduced alveolar oedema, congestion, and neutrophil infiltration [18, 57]. Similar benefits have been derived from hepatosplanchnic conditioning, which abrogated the P-selectin upregulation caused by IRI, with an attendant reduction in alveolar and perivascular neutrophil infiltration, reduced MDA levels, and preserved vascular permeability [58]. Likewise, hepatic or mesenteric conditioning reduced inflammatory cell infiltration in animals undergoing hepatosplanchnic reperfusion [59]. In concordance with these findings, Meng et al*.* showed that mesenteric conditioning decreased plasma and lung tissue TNF-a and IL-6 levels, in contrast with an increase in IL-10 levels, SOD and glutathione peroxidase activity. Moreover, endothelial and alveolar epithelial architecture were preserved, as was the integrity of type II alveolar cell mitochondria, with concurrently diminished wet-to-dry lung weight ratio and pulmonary microvascular dysfunction [60]. However, dos Santos et al*.* did not reveal any histologic preservation by IC; interestingly, mesenteric IRI caused only minimal lung injury, thereby narrowing the margins for demonstrating a protective effect [61].

Harkin et al*.* investigated the effects of IC on limb reperfusion-induced LIRI, by way of external iliac artery occlusion/reperfusion. IC attenuated the increase in plasma IL-6 and phagocytic priming, without affecting the TNF-a levels; lung tissue MPO increase was also diminished, as was the elevation of weight-to-dry weight ratio [62]. A similar study focused on unilateral lower limb ischaemia, utilising a left lower limb tourniquet. RIC resulted in decreased plasma TBARS, as well as reduced PMN infiltration and MPO activity in the lung, while protecting from alveolar and interstitial oedema, and alveolar haemorrhage [63].

Haemorrhagic shock resuscitation is hampered by multiorgan failure (MOF), which is the commonest cause of death after severe trauma [64]. The role of systemic inflammation and oxidative stress is pivotal [65], while respiratory failure is recognised among the commonest and deadliest complications thereof [64]. Jan et al. studied the effects of lower limb tourniquet occlusion/reperfusion on a rat model of haemorrhagic shock. IC significantly reduced plasma IL-6 levels, in parallel with diminished lung IL-6, PGE2, MDA, and BAL fluid protein concentration. The levels of macrophage inflammatory protein-2 (MIP-2), MPO activity, PMN-to-alveoli and wet-to-dry weight ratios were also decreased, in association with mitigated alveolar wall oedema, haemorrhagic changes, vascular congestion, and PMN infiltration [66]. In a comparable study, RIC inhibited the rise in plasma TNF-a levels, as well as the lung TNF-a mRNA and protein expression following shock resuscitation. Lung MPO activity and protein leakage into BAL fluid were similarly reduced [67].

The suspension of ventilation, in concert with the resultant HPV, result in a hypoxic and hypoperfused lung environment that instigates LIRI [3, 5]. Preliminary evidence in this context have been contradictory. Bergmann et al*.* exploited RIC in a swine model of one lung ventilation (OLV). On the one hand, lung TNF-a and BAL fluid leucocyte levels were reduced; on the other hand, serum IL-1β and IL-8 were not affected, while microhaemorrhage and alveolar oedema of the ventilated lung were enhanced [68].

**Fig. 2:**
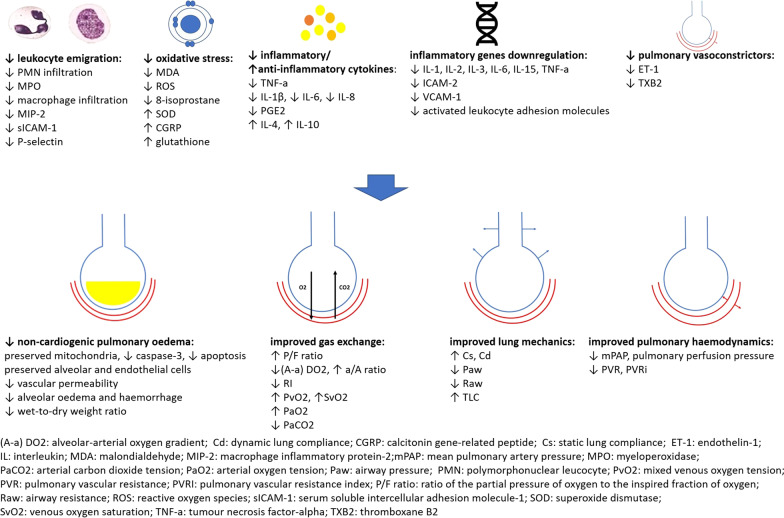
The protective effects of ischaemic conditioning from lung ischaemia reperfusion injury. Ischaemic conditioning mitigates leukocyte migration, oxidative stress and systemic inflammatory cascades, while circulating inflammatory cytokine and pulmonary vasoconstrictor levels are also reduced. These mechanisms culminate in the amelioration of alveolar and endothelial injury, resulting in protection from non-cardiogenic pulmonary oedema, improved gas exchange, improved lung mechanics and superior pulmonary haemodynamics

The marked heterogenicity of experimental IC protocols obviates reaching a safe conclusion with regards to their differential efficacy. Up to six cycles of ischaemia/reperfusion, with individual cycle ischaemic durations ranging from 10 s to 15 min have been successfully utilised. Thus, it may be inferred that a cumulative ischaemic stimulus of 1 and up to 30 min may confer biochemical protection from LIRI, although the most commonly applied protocols comprised three or four 5- or 10-min ischaemia–reperfusion cycles (Tables 1, 3; Fig. 2).

**Table 1:** Experimental studies of the ischaemic conditioning/remote ischaemic conditioning effects on lung ischaemia reperfusion injury

	Model	Ischaemic conditioning protocol	Oxidative stress and inflammation	Respiratory function and pulmonary haemodynamics
Song et al. [17]	Rat lung in situ	Six aortic 10-s/10-s cycles	Reduced plasma IL-6, TNF-a, ROS, reduced alveolar PMN infiltration and oedema, reduced lung wet-to-dry ratio	Increased PaO2, reduced PaCO2
Dorsa et al. [18]	Rat abdominal aortic occlusion	Three 2-min/2-min abdominal aortic cycles	Reduced alveolar oedema, congestion, and PMN infiltration	NA
Luo et al. [19]	Rat lung in situ	Four 5-min/5 min hepatic hilar cycles	Reduced plasma IL-6 and TNF-a, reduced lung MPO activity and BAL fluid WBC count, reduced alveolar damage and lung wet-to-dry ratio, reduced lung caspase-3 expression and apoptotic nuclei	Increased PaO2, reduced PaCO2
Waldow et al. [20]	Porcine lung in situ	Three 5-min/5-min femoral arterial cycles	Reduced plasma IL-1β and macrophage count, reduced lung macrophage infiltration, plasma IL-6 and ROS not affected	Increased PaO2 and PvO2, reduced PAP and PVR
Li et al. [48]	Canine lung transplantation’	One 10-min-15-min donor lung cycle	Reduced lung PMN infiltration, reduced serum MDA and TXB2, increased SOD	Increased PvO2, reduced mPAP
Du et al. [49]	Rat lung transplantation	One 5 min/10-min donor lung cycle	Reduced TBARS	Increased PaO2, decreased PaCO2
Gasparri et al. [50]	Rabbit lung preservation	One lung 5-min/10-min vs three 5-min/10-min vs five 3-min/6-min cycles	Reduced lung oedema	Increased veno-arterial PO2 gradient observed following the conditioning protocols of 15-min total ischaemic stimulus
Soncul et al. [51]	Guinea pig lung preservation	Two lung 5-min/5 min cycles	Reduced lung tissue and perfusate MDA, reduced glutathione	Reduced PAP
Kandilci et al. [52]	Rat lung preservation	Two lung 5-min/5 min cycles	Reduced tissue MDA and glutathione, reduced intra-alveolar oedema and capillary congestion, type II alveolar and endothelial cell preservation	Reduced PAP
Li et al. [53]	Rabbit lung in situ	One lung 10-min/15-min cycle	Reduced lung MDA, lung oedema and alveolar damage, increased lung SOD	Increased PaO2, decreased mPAP
Friedrich et al. [54]	Canine lung in situ	One lung 5-min/15-min vs two 10-min/10-min cycles	Reduced BAL fluid protein and TNF-a following the 5-min ischaemic stimulus conditioning protocol	Increased PaO2 and PvO2, increased Cd, following the 5-min ischaemic stimulus conditioning protocol; neutral effects on PVR following both protocols
Jun et al. [55]	Rat lung transplantation	Three donor lung 5-min/5-min cycles	Inflammatory and immune mediator genes downregulation	NA
Zhou et al. [56]	Rat cardiopulmonary bypass	Three 5-min/5-min hind limb cycles	Reduced alveolar PMN infiltration and wall thickening, reduced BAL fluid protein, reduced lung wet-to-dry ratio, increased serum IL-4 and -10	Increased TLC and Cd, reduced Raw
Akahane et al. [57]	Rat abdominal aortic occlusion	Three 2-min/2-min abdominal aortic cycles	Reduced lung PMN infiltration and interstitial oedema	NA
Peralta et al. [58]	Rat hepatic ischaemia–reperfusion	One 10-min/10-min hepatic cycle	Reduced P-selectin upregulation, reduced lung PMN infiltration and MDA, preserved vascular permeability	NA
Neto et al. [59]	Rat hepatic/splanchnic ischaemia–reperfusion	One 10-min/10-min hepatic hilar/mesenteric arterial cycle	Reduced lung inflammatory cell infiltration	NA
Meng et al. [60]	Mouse splanchnic ischaemia–reperfusion	Three 30-s/30-s mesenteric arterial cycles	Reduced plasma and lung TNF-a and IL-6, increased plasma and lung IL-10, increased lung SOD and glutathione peroxidase activity, preserved endothelial and alveolar architecture	NA
Dos Santos et al. [61]	Rat splanchnic ischaemia–reperfusion	Two 2-min/2-min vs four 30-s/30-s mesenteric arterial cycles	Neutral effects on histological findings	NA
Harkin et al. [62]	Porcine limb ischaemia–reperfusion	Three 5-min/5-min external iliac arterial cycles	Reduced plasma IL-6 and phagocytic priming, reduced lung MPO and wet-to-dry weight ratio, neutral effect on plasma TNF-a	Increased PaO2, reduced (A-a) DO2, reduced mPAP
Olguner et al. [63]	Rat lower limb ischaemia–reperfusion	Three 10-min/10-min lower limb tourniquet cycles	Reduced plasma TBARS, reduced lung PMN infiltration and MPO activity, reduced lung oedema and alveolar haemorrhage	NA
Jan et al. [66]	Rat haemorrhagic shock	Three 10-min/10-min lower limb tourniquet cycles	Reduced plasma IL-6, reduced lung IL-6, PGE2, and MDA, reduced BAL fluid protein, reduced lung MIP-2, MPO activity, PMN-to-alveoli and wet-to-dry weight ratio, reduced histological injury	Increased PaO2, reduced (A-a) DO2, increased pH and BE
Leung et al. [67]	Mouse haemorrhagic shock	Four 5-min/5-min lower limb tourniquet cycles	Reduced plasma TNF-a, reduced lung TNF-a mRNA and protein expression, reduced lung MPO activity and BAL fluid protein	NA
Bergmann et al. [68]	Swine one lung ventilation	Three 5-min/5-min hind limb tourniquet cycles	Reduced lung TNF-a and BAL fluid WBC, neutral effect on serum IL-1β and -8, enhanced lung microhaemorrhage and alveolar oedema	Reduced oxygenation index, increased SvO2
Featherstone et al. [75]	Rat lung preservation	One lung 5-min/5-min vs one 10-min/5-min vs two 5-min/5-min lung cycles	NA	Increased lung compliance, neutral effects on oxygenation and PVR observed following all conditioning protocols
Kharbanda et al. [76]	Swine cardiopulmonary bypass	Four 5-min/5-min hind limb tourniquet cycles	NA	Reduced Raw and ventilation pressures, neutral effect on PVR
Xia et al. [77]	Sheep coronary arterial occlusion	Three 5-min/5-min iliac arterial cycles	NA	Increased PaO2 and P/F ratio, decreased PVR and PAP

*(A-a) DO2* alveolar-arterial oxygen gradient, *BAL* bronchoalveolar lavage, *Cd* dynamic lung compliance, *IC* Ischaemic conditioning, *IL* interleukin, *IRI* ischaemia–reperfusion injury, *MDA* malondialdehyde, *MIP-2* macrophage inflammatory protein-2, *mPAP* mean pulmonary artery pressure, *MPO* myeloperoxidase, *NA* not applicable, *PaCO2* arterial carbon dioxide tension, *PaO2* arterial oxygen tension, *PAP* pulmonary artery pressure, *PMN* polymorphonuclear leucocyte, *PvO2* mixed venous oxygen tension, *PVR* pulmonary vascular resistance, *P/F ratio* ratio of the partial pressure of oxygen to the inspired fraction of oxygen, *Raw* airway resistance, *RIC* remote ischemic conditioning, *ROS* reactive oxygen species, *SOD* superoxide dismutase, *SvO2* venous oxygen saturation, *TBARS* thiobarbituric acid reactive substances, *TLC* total lung capacity, *TNF-a* tumour necrosis factor-alpha, *TXB2* thromboxane B2

### Clinical evidence

The majority of clinical data pertaining to the investigation of IC effects on LIRI has been obtained in the context of CPB-induced IRI. Forty patients undergoing valve replacement were randomised to an IC or control group. Analysis of pulmonary vein blood showed that IC by way of aortic occlusion/reperfusion mitigated the increase in MDA, PMN, and TBX_2_ levels, while increasing SOD. Similarly, calcitonin gene-related peptide (CGRP) levels in coronary sinus blood were enhanced, denoting the activation of the associated anti-oxidant pathway, in parallel with reduced pulmonary oedema, haemorrhage, and PMN infiltration [10]. RIC has similar properties, as exemplified by Jin et al*.* who utilised upper limb and thigh cuff inflation/deflation in a randomised control trial (RCT) of 241 patients undergoing valvular replacement: conditioning reduced the levels of serum soluble intercellular adhesion molecule-1 (sICAM-1), endothelin-1 (ET-1), and MDA, while increasing NO concentration [69]. In an RCT of 60 infants undergoing ventricular septal defect (VSD) repair, Zhou et al*.* applied upper limb cuff inflation/deflation in the intervention group. Postoperatively, serum IL-6, -8, -10, and TNF-a levels were reduced, while coronary sinus MDA was reduced and SOD increased in preconditioned infants [12]. Nonetheless, these findings are not unequivocal. Lower limb cuff inflation/deflation was applied to children undergoing surgical repair of congenital heart defects, but the realised lung protection could not be associated with systemic inflammation, as IL-6, -8, -10, and TNF-a levels were not affected by RIC [70]. Similarly, Hu et al. conducted an RCT that included 201 patients undergoing valve replacement, whereby RIC did not affect hypersensitive C-reactive protein (hsCRP) levels [11].

IC has been utilised in patients undergoing thoracic surgical operations. Chen et al*.* have been among the first to implement preconditioning in patients undergoing pneumonectomy: in a small study of 20 patients, a single pulmonary artery occlusion/reperfusion manoeuvre resulted in increased CGRP and SOD levels [71]. Li et al*.* randomised 216 patients undergoing elective lung resection to either a RIC, or a standard thoracic surgical treatment arm. Upper limb cuff inflation/deflation significantly reduced IL-6, TNF-a, and MDA levels post-operatively [7]. An analogous RCT was performed in 55 patients undergoing lobectomy for lung cancer, whose blood and exhaled breath condensate (EBC) were examined to determine the levels of oxidative stress. RIC mitigated oxidative stress, as denoted by reduced 8-isoprostane, cumulative nitrate and nitrite, hyperoxide, and acidity in the EBC samples of preconditioned patients; blood 8-isoprostane as well as cumulative nitrate and nitrite were also reduced in the RIC arm [8]. These findings were not reproduced in the pilot RCT by Lin et al*.* who randomised 60 patients undergoing bilateral sequential lung transplantation to a RIC or a standard treatment arm: IL-2, -6, -8, -10, TNF-a, interferon-gamma (IFN-γ), interferon gamma-induced protein 10, monocyte chemoattractant protein-1 (MCP-1), and chemokine ligand 5 (CCL5) did not differ between the two study groups [72].

Aortic cross-clamping during open abdominal aortic aneurysm repair (AAA) is known to trigger a systemic inflammatory response with an attendant accentuation of oxidative stress [33]; these cascades may progress to MOF, which underlies up to 25% of peri-operative deaths in this setting [73]. Against this background, the effects of RIC in the form of upper limb cuff inflation/deflation were evaluated in an RCT of 62 patients undergoing open AAA repair. RIC attenuated the post-operative increase in IL-6, TNF-a, and MDA levels, while increasing the activity of SOD [13]. Limb reperfusion may also induce MOF through comparable mechanisms [74]. Lin et al*.* studied 30 patients undergoing lower extremity surgery, necessitating sustained thigh tourniquet application. When this was preceded by IC, the rise in plasma IL-6, -8, and MDA after limb reperfusion was mitigated [14].

Similar to experimental investigations, clinical IC protocols have not been standardised. However, three 5-min ischaemia–reperfusion cycles have been most commonly utilised and may confer a biochemically determined protection from LIRI in a variety of clinical settings, although some studies have shown a neutral effect (Tables 2, 3; Fig. 2).

**Table 2:** Clinical studies of the ischaemic conditioning/remote ischaemic conditioning effects on lung ischaemia reperfusion injury

Study	Clinical setting	Ischaemic conditioning protocol	Oxidative stress and inflammation	Respiratory function and pulmonary haemodynamics
Li et al. [7]	Lung resection	Three 5-min/5-min arm cuff cycles	Reduced serum IL-6, TNF-a, and MDA	Increased P/F and a/A ratio, reduced (A-a) DO2, reduced ALI incidence, increased Cs and Cd
García-de-la-Asunción et al. [8]	Lobectomy	Three 5-min/5-min arm cuff cycles	Reduced EBC 8-isoprostane, nitrates and nitrites, hyperoxide, and acidity, reduced blood 8-isoprostane, nitrates and nitrites, neutral effect on CRP	Increased PaO2, P/F and a/A ratio, decreased (A-a) DO2 and RI
Li et al. [10]	Valve replacement (cardiopulmonary bypass)	Two 3-min/2-min aortic cycles	Reduced pulmonary vein MDA, PMN, and TBX2, increased SOD, increased coronary sinus CGRP, reduced lung oedema, haemorrhage, and PMN infiltration	Reduced PVRI and mPAP, increased PaO2, reduced pulmonary complications (atelectasis, pneumonitis, pneumothorax)
Hu et al. [11]	Valve replacement (cardiopulmonary bypass)	Three 5-min/5-min thigh cycles	Neutral effect on serum hsCRP	Reduced ALI incidence, neutral effect on A-aO2
Zhou et al. [12]	Infantile ventricular septal defect repair (cardiopulmonary bypass)	Three 5-min/5-min arm cuff cycles	Reduced serum IL-6, -8, -10, and TNF-a, reduced coronary sinus MDA, increased coronary sinus SOD	Reduced RI, increased Cs and Cd
Li et al. [13]	Abdominal aortic aneurysm repair	Three 5-min/5-min arm cuff cycles	Reduced plasma IL-6, TNF-a, and MDA, increased SOD	Increased a/A ratio, reduced (A-a) DO2 and RI, increased Cs and Cd
Lin et al. [14]	Lower limb surgery	Three 5-min/5-min thigh tourniquet cycles	Reduced plasma IL-6, -8, and MDA	Increased PaO2 and a/A ratio, reduced (A-a) DO2 and RI
Jin et al. [69]	Valve replacement (cardiopulmonary bypass)	Two 5-min/5-min arm and thigh cuff cycles	Reduced serum sICAM-1, ET-1, and MDA, increased NO	Reduced (A-a) DO2, RI, ALI incidence
Cheung et al. [70]	Children congenital heart defect repair (cardiopulmonary bypass)	Four 5-min/5-min thigh cuff cycles	Neutral effect on serum IL-6, -8, -10, and TNF-a	Reduced Paw, neutral effect on oxygenation and compliance
Chen et al. [71]	Pneumonectomy	One 10-min/10-min pulmonary arterial cycle	Increased serum CGRP and SOD	Increased PvO2
Lin et al. [72]	Lung transplantation	Three 5-min/5-min lower limb cuff cycles	Neutral effect on IL-2, -6, -8, -10, TNF-a, IFN-γ, interferon gamma-induced protein 10, MCP-1, and CCL5	Trend for decreased PGD and biopsy-proven rejection risk, increased P/F ratio in restrictive lung disease group
Min et al. [78]	Valve replacement (cardiopulmonary bypass)	Four 5-min/5-min arm cuff cycles	NA	Increased P/F ratio, reduced need for mechanical ventilation > 48 h, neutral effect on Cs and Cd
Hong et al. [79]	Off-pump coronary artery bypass grafting	Four 5-min/5-min lower limb cuff cycles applied twice	NA	Neutral effect on oxygenation and duration of mechanical ventilation
Kim et al. [80]	Valvular heart surgery (cardiopulmonary bypass)	Three 10-min/10-min lower limb cuff cycles applied twice	NA	Neutral effect on respiratory function and outcomes
Rahman et al. [81]	Coronary artery bypass grafting (cardiopulmonary bypass)	Three 5-min/5-min arm cuff cycles	NA	Neutral effect on respiratory function and outcomes
Lee et al. [82]	Infantile ventricular septal defect repair (cardiopulmonary bypass)	Four 5-min/5-min thigh cuff cycles	NA	Neutral effect on respiratory function and outcomes

*ALI* acute lung injury, *(A-a) DO2* alveolar-arterial oxygen gradient, *CCL5* chemokine ligand 5, *Cd* dynamic lung compliance, *CGRP* calcitonin gene-related peptide, *Cs* static lung compliance, *EBC* exhaled breath condensate, *ET-1* endothelin-1, *hsCRP* hypersensitive C-reactive protein, *IC* Ischaemic conditioning, *IFN-γ* interferon-gamma, *IL* interleukin, *IRI* ischaemia–reperfusion injury, *MCP-1* monocyte chemoattractant protein-1, *MDA* malondialdehyde, *mPAP* mean pulmonary artery pressure, *NA* not applicable, *PaO2* arterial oxygen tension, *PAP* pulmonary artery pressure, *Paw* airway pressure, *PGD* primary graft dysfunction, *PMN* polymorphonuclear leucocyte, *PvO2* mixed venous oxygen tension, *PVRI* pulmonary vascular resistance index, *P/F ratio* ratio of the partial pressure of oxygen to the inspired fraction of oxygen, *Raw* airway resistance, *RIC* remote ischemic conditioning, *sICAM-1* serum soluble intercellular adhesion molecule-1, *SOD* superoxide dismutase, *TNF-a* tumour necrosis factor-alpha

**Table 3:** Summary of the protective effects of ischaemic conditioning from lung ischaemia–reperfusion injury

Ischaemic conditioning protective effects from lung ischaemia reperfusion injury	Studies
↓ Leukocyte emigration	Li et al. [10]; Song et al. [17]; Dorsa et al. [18]; Luo et al. [19]; Waldow et al. [20]; Li et al. [48]; Zhou et al. [56]; Akahane et al. [57]; Peralta et al. [58]; Neto et al. [59]; Harkin et al. [62]; Olguner et al. [63]; Jan et al. [66]; Leung et al. [67]; Bergmann et al. [68]; Jin et al. [69]
↓ Oxidative stress	Li et al. [7]; García-de-la-Asunción et al. [8]; Li et al. [10]; Zhou et al. [12]; Li et al. [13]; Lin et al. [14]; Song et al. [17]; Li et al. [48]; Du et al. [49]; Soncul et al. [51]; Kandilci et al. [52]; Li et al. [53]; Peralta et al. [58]; Meng et al. [60]; Olguner et al. [63]; Jan et al. [66]; Jin et al. [69]; Chen et al. [71];
↓ Inflammatory/ ↑Anti-inflammatory cytokines	Zhou et al. [12]; Li et al. [7]; Li et al. [13]; Lin et al. [14]; Song et al. [17]; Luo et al. [19]; Waldow et al. [20]; Zhou et al. [56]; Meng et al. [60]; Leung et al. [67]; Bergmann et al. [68]
Inflammatory genes downregulation	Jun et al. [55]
↓ Pulmonary vasoconstrictors	Li et al. [10]; Li et al. [48]; Jin et al. [69]
↓ Non-cardiogenic pulmonary oedema	Li et al. [10]; Song et al. [17]; Dorsa et al. [18]; Luo et al. [19]; Gasparri et al. [50]; Kandilci et al. [52]; Li et al. [53]; Friedrich et al. [54]; Zhou et al. [56]; Akahane et al. [57]; Peralta et al. [58]; Meng et al. [60]; Harkin et al. [62]; Olguner et al. [63]; Jan et al. [66]
Improved gas exchange	Li et al. [7]; García-de-la-Asunción et al. [8]; Li et al. [10]; Zhou et al. [12]; Li et al. [13]; Lin et al. [14]; Song et al. [17]; Luo et al. [19]; Waldow et al. [20]; Li et al. [48]; Du et al. [49]; Li et al. [53]; Friedrich et al. [54]; Harkin et al. [62]; Jan et al. [66]; Bergmann et al. [68]; Jin et al. [69]; Chen et al. [71]; Lin et al. [72]; Xia et al. [77]; Min et al. [78]
Improved lung mechanics	Li et al. [7]; Zhou et al. [12]; Li et al. [13]; Friedrich et al. [54]; Cheung et al. [70]; Featherstone et al. [75]; Kharbanda et al. [76]
Improved pulmonary haemodynamics	Li et al. [10]; Waldow et al. [20]; Li et al. [48]; Soncul et al. [51]; Kandilci et al. [52]; Li et al. [53]; Harkin et al. [62]; Xia et al. [77]

## The protective effects of ischemic conditioning on lung ischaemia reperfusion injury—effects on respiratory function and pulmonary haemodynamics

### Experimental evidence

The oxidative stress and inflammatory response alleviation conferred by IC may be functionally translated into improved respiratory function and pulmonary haemodynamics. In the canine transplantation model studied by Li et al*.* donor lung IC was associated with increased mixed venous oxygen tension (PvO2) and reduced mean pulmonary artery pressure (mPAP) following reperfusion [48]. Du et al*.* also provided evidence of improved gas exchange, as denoted by higher arterial oxygen (PaO_2_) and decreased carbon dioxide tension (PaCO_2_) levels [49]. Similar oxygenation improvement has been demonstrated in the form of increased veno-arterial oxygen pressure gradients [50], while Soncul et al*.* concluded that IC ameliorated the increase in pulmonary artery pressure (PAP) caused by LIRI [51]. IC also preserved pulmonary arterial endothelial function in the study of Kandilci et al., reflected on decreased pulmonary perfusion pressures in response to histamine [52]. Further expanding the evidence above, Li et al. showed that PO2 is increased and mPAP decreased by IC [53]. Data of improved Cd have also been provided, in parallel with higher PaO2 and PvO2 levels; however, in the same study pulmonary vascular resistance (PVR) was not significantly affected [54]. Featherstone et al*.* reported analogous improvement in lung compliance; nonetheless, oxygenation and PVR did not differ between the preconditioned and the control groups [75].

RIC exerts comparable protective effects: gas exchange was significantly improved in the context of an in situ LIRI model, with increased PaO2 and decreased PaCO2, in the studies of Luo et al. [19] and Song et al. [17]. Waldow et al*.* showed that RIC improved PaO2 and PvO2, in parallel with a reduction in PAP and PVR [20]. In a setting of CPB-induced LIRI, evidence of superior total lung capacity (TLC) and Cd, as well as reduced airway resistance (Raw) was provided [56]. Comparably, Raw was lower upon exposure to CPB with a resultant decrease in ventilation pressures and a trend for lung compliance improvement in the study of Kharbanda et al. [76]. In an interesting study of coronary occlusion/reperfusion, simulating an off-pump coronary artery bypass stimulus, RIC increased PaO2 and P/F ratio, while decreasing PVR and PAP [77].

Investigating LIRI within the realms of remote reperfusion injury, Harkin et al. showed an improvement in PaO2 and (A-a) DO2 with an attendant reduction in mPAP following limb reperfusion in preconditioned animals [62]. Jan et al*.* demonstrated similar protective properties after resuscitation from haemorrhagic shock, with increased PaO2 and decreased (A-a) DO2, with an attendant amelioration of acidosis, as pH and base excess were increased by IC [66]. Interestingly, Bergmann et al. highlighted the improved respiratory function conferred by IC during OLV: oxygenation index [fraction of inspired oxygen (FIO2) * mean airway pressure (Paw mean)/PaO2)] was lower after RIC, with an attendant increase in venous oxygen saturation (SvO2) [68].

In summary, most experimental studies have reported improvements in gas exchange, respiratory mechanics, and pulmonary haemodynamics by IC. Interestingly, local conditioning appears to exert similar effects to RIC, both in settings of in situ as well as remote reperfusion injury. Thus, the systemic nature of the underlying processes has been consistently demonstrated (Tables 1, 3; Fig. 2).

### Clinical evidence

There is accumulating clinical evidence delineating the effects of IC against LIRI in several pathophysiological contexts. Li et al. investigated the application if IC in patients exposed to CPB and revealed that preconditioning reduced PVRI and mPAP, while increasing PaO2; importantly, there was an attendant reduction in pulmonary complications (lobar collapse, pneumonitis, pneumothorax) and mechanical ventilation time [10]. In a similar cohort of patients, RIC conferred a decrease in (A-a) DO2, respiratory index [(A-a) DO2/PaO2], as well as the incidence of ALI [69]. Zhou et al. also concluded that RIC improved RI and decreased static (Cs) and dynamic lung compliance (Cd) [12], while the incidence of ALI was reduced in patients undergoing RIC during their valve replacement, despite the fact that (A-a) DO2 was not affected [11]. Moreover, RIC applied both before and after CBP in patients undergoing valvular replacement improved P/F ratio and reduced the incidence of mechanical ventilation beyond 48 h; Cs and Cd remained unchanged [78]. However, Cheung et al*.* did not reproduce any improvements in either oxygenation or compliance; nonetheless, Raw was reduced in preconditioned patients [70]. Hong et al. also showed a neutral effect of RIC on oxygenation and mechanical ventilation duration; however, the included patients underwent off-pump CABG, which obviates the exposure to CPB [79]. Additional studies have shown a lack of improvement in respiratory indices or outcomes, but no inflicted harm [80–82].

The results of the studies focusing on patients undergoing lung resections uniformly support the notion that IC confers significant protection from LIRI. RIC abrogated the deleterious effects of OLV on respiratory function, as denoted by improvements in P/F ratio, (A-a) DO2, and arterial-alveolar oxygen tension ratio (a/A ratio), with an attendant decreased ALI incidence; Cs and Cd were also significantly increased [7]. Concordant evidence was provided by García-de-la-Asunción et al., who showed an improvement in PaO2, (A-a) DO2, P/F and a/A ratio, and RI [8]. Chen et al*.* also demonstrated increased pulmonary venous oxygen tension in patients undergoing pneumonectomy [71]. In an important pilot RCT of RIC within the context of lung transplantation by Lin et al., preconditioning was associated with a trend for decreased PGD and biopsy-proven rejection risk, while P/F ratio was significantly increased in the subgroup of patients with restrictive lung disease [72]. RIC protects from the lung dysfunction triggered by splanchnic IRI, as shown by the RCT of Lin et al., whereby preconditioned patients undergoing AAA repair benefited from higher a/A ratio, lower (A-a) DO2 and RI, as well as improved Cs and Cd [13]. Comparable effects have been demonstrated following lower limb reperfusion: RIC resulted in improved PaO2, a/A ratio, (A-a) DO2, and RI [14].

Thus, despite the contradictory evidence provided by studies of cardiac surgery patients, the majority of data obtained in clinical settings that entail in situ lung, as well as hepatosplanchnic and limb reperfusion underlines the beneficial effects obtained from IC: respiratory function and ventilation mechanics may improve, while respiratory complications may be averted (Tables 2, 3; Fig. 2).

## Conclusions

LIRI has a detrimental effect on respiratory function and pulmonary haemodynamics, with dismal consequences for patient prognosis in a variety of clinical settings. The abundance of experimental evidence revealing the beneficial effects of IC, both on the underlying inflammatory and oxidative cascades, as well as the resultant functional derangements is being gradually complemented by encouraging clinical data. Given its promising preliminary results, safety, and ease of application, IC appears to be an intervention worth of further investigation and a useful addition to our deficient armamentarium against LIRI.

## Data Availability

Not applicable.

## Abbreviations

AAA: Abdominal aortic aneurysm
ALI: Acute lung injury
ARDS: Acute respiratory distress syndrome
ATP: Adenosine triphosphate
(A-a) DO2: Alveolar-arterial oxygen gradient
BAL: Bronchoalveolar lavage
CCL5: Chemokine ligand 5
Cd: Dynamic lung compliance
CGRP: Calcitonin gene-related peptide
CPAP: Continuous positive airway pressure
Cs: Static lung compliance
EBC: Exhaled breath condensate
ET-1: Endothelin-1
FIO2: Fraction of inspired oxygen
HPV: Hypoxic pulmonary vasoconstriction
hsCRP: Hypersensitive C-reactive protein
IC: Ischaemic conditioning
IFN-γ: Interferon-gamma
IL: Interleukin
IRI: Ischaemia–reperfusion injury
LIRI: Lung ischaemia–reperfusion injury
MCP-1: Monocyte chemoattractant protein-1
MDA: Malondialdehyde
MIP-2: Macrophage inflammatory protein-2
MOF: Multiorgan failure
mPAP: Mean pulmonary artery pressure
MPO: Myeloperoxidase
OLV: One lung ventilation
PaCO2: Arterial carbon dioxide tension
PaO2: Arterial oxygen tension
PAP: Pulmonary artery pressure
Paw: Airway pressure
PEEP: Positive end-expiratory pressure
PGD: Primary graft dysfunction
PMN: Polymorphonuclear leucocyte
PvO2: Mixed venous oxygen tension
PVR: Pulmonary vascular resistance
PVRI: Pulmonary vascular resistance index
P/F ratio: Ratio of the partial pressure of oxygen to the inspired fraction of oxygen
Raw: Airway resistance
RCT: Randomised control trial
RIC: Remote ischemic conditioning
ROS: Reactive oxygen species
sICAM-1: Serum soluble intercellular adhesion molecule-1
SOD: Superoxide dismutase
SvO2: Venous oxygen saturation
TBARS: Thiobarbituric acid reactive substances
TLC: Total lung capacity
TNF-a: Tumour necrosis factor-alpha
TXB2: Thromboxane B2
VCAM-1: Vascular cell adhesion molecule-1
VSD: Ventricular septal defect
V/Q mismatch: Ventilation-perfusion mismatch
